# Supplementary material for: Assisted quantum simulation of open quantum systems
Source: iScience. 2023 Mar 3;26(4):106306. doi: 10.1016/j.isci.2023.106306 (PMC10040711; doi:10.1016/j.isci.2023.106306)
Supplement: Document S1. Table S1 [file mmc1.pdf]

iScience, Volume *26*

Supplemental information

Assisted quantum simulation of open quantum systems

Jin-Min Liang, Qiao-Qiao Lv, Zhi-Xi Wang, and Shao-Ming Fei

Table S1: The LCU decomposition of the non-Hermitian Hamiltonian, related to STAR Methods.

k	0	1	2	3	4
$\widehat{\mathcal{H}}_k$	$\iota\sigma_z^{(3)}\otimes\sigma_z^{(4)}$	$\iota\sigma_x^{(3)}$	$\iota\sigma_x^{(4)}$	$-\iota\sigma_z^{(1)}\otimes\sigma_z^{(2)}$	$-\iota\sigma_x^{(1)}$
k	5	6	7	8	9
$\widehat{\mathcal{H}}_k$	$-\iota\sigma_x^{(2)}$	$\sigma_x^{(1)}\otimes\sigma_x^{(3)}$	$-\iota\sigma_x^{(1)}\otimes\sigma_y^{(3)}$	$-\iota\sigma_y^{(1)}\otimes\sigma_x^{(3)}$	$-\sigma_y^{(1)}\otimes\sigma_y^{(3)}$
k	10	11	12	13	14
$\widehat{\mathcal{H}}_k$	$-\iota\sigma_z^{(3)}$	$-\sigma_z^{(1)}$	$\sigma_x^{(2)}\otimes\sigma_x^{(4)}$	$-\iota\sigma_x^{(2)}\otimes\sigma_y^{(4)}$	$-\iota\sigma_y^{(2)}\otimes\sigma_x^{(4)}$
k	15	16	17	18	
$\widehat{\mathcal{H}}_k$	$-\sigma_y^{(2)}\otimes\sigma_y^{(4)}$	$-\sigma_z^{(4)}$	$-\sigma_z^{(2)}$	$-I_{16}$	

The unitary operators of the non-Hermitian Hamiltonian $\widehat{\mathcal{H}}$ in the LCU decomposition.
